# Can Carbohydrate Mouth Rinse Improve Performance during Exercise? A Systematic Review

**DOI:** 10.3390/nu6010001

**Published:** 2013-12-19

**Authors:** Thays de Ataide e Silva, Maria Eduarda Di Cavalcanti Alves de Souza, Jamile Ferro de Amorim, Christos G. Stathis, Carol Góis Leandro, Adriano Eduardo Lima-Silva

**Affiliations:** 1 Sport Science Research Group, Department of Physical Education and Sports Science (CAV), Federal University of Pernambuco, Alto do Reservatório street, Bela Vista, Vitória de Santo Antão, Pernambuco 55608-680, Brazil; E-Mails: thays_de_ataide@hotmail.com (T.A.S.); dicavalcanti@hotmail.com.br (M.E.C.A.S.); jamiferro@hotmail.com (J.F.A.); 2 Laboratory of Physiology and Pharmacology, Department of Physical Education and Sports Science (CAV), Federal University of Pernambuco, Alto do Reservatório street, Bela Vista, Vitória de Santo Antão, Pernambuco 55608-680, Brazil; E-Mail: carolleandro22@gmail.com; 3 College of Health and Biomedicine and Institute of Sport Exercise and Active Living (iSEAL), Victoria University, Melbourne 8001, Australia; E-Mail: christos.stathis@vu.edu.au

**Keywords:** maltodextrin, glucose, mouthwash, performance

## Abstract

The purpose of this review was to identify studies that have investigated the effect of carbohydrate (CHO) mouth rinse on exercise performance, and to quantify the overall mean difference of this type of manipulation across the studies. The main mechanisms involving the potential benefit of CHO mouth rinse on performance was also explored. A systematic review was conducted in the following electronic databases: PubMed, SciELO, Science Direct, MEDLINE, and the Cochrane Library (Cochrane Central Register of Controlled Trials), without limit of searches. Eleven studies were classified as appropriate and their results were summarized and compared. In nine of them, CHO mouth rinse increased the performance (range from 1.50% to 11.59%) during moderate- to high-intensity exercise (~75% Wmax or 65% VO_2_max, ~1 h duration). A statistical analysis to quantify the individual and overall mean differences was performed in seven of the 11 eligible studies that reported power output (watts, W) as the main performance outcome. The overall mean difference was calculated using a random-effect model that accounts for true variation in effects occurring in each study, as well as random error within a single study. The overall effect of CHO mouth rinse on performance was significant (mean difference = 5.05 W, 95% CI 0.90 to 9.2 W, *z* = 2.39, *p* = 0.02) but there was a large heterogeneity between the studies (*I*^2^ = 52%). An activation of the oral receptors and consequently brain areas involved with reward (insula/operculum frontal, orbitofrontal cortex, and striatum) is suggested as a possible physiological mechanism responsible for the improved performance with CHO mouth rinse. However, this positive effect seems to be accentuated when muscle and liver glycogen stores are reduced, possibly due to a greater sensitivity of the oral receptors, and require further investigation. Differences in duration of fasting before the trial, duration of mouth rinse, type of activity, exercise protocols, and sample size may account for the large variability between the studies.

## 1. Introduction

Carbohydrate (CHO) mouth rinse is defined as a CHO fluid distribution around the mouth for 5 to 10 s with subsequent expulsion by spitting. The utilization of either a low-concentrated 6.0% to 6.4% glucose [1] or partially hydrolyzed maltodextrin are the most common CHO used, with the latter being colorless and tasteless when dissolved in water [2]. CHO mouth rinse has been investigated as a potential ergogenic resource for improved performance during moderate- to high-intensity exercises (~75% VO_2_max, ~1 h duration). Carter *et al.* [3] first studied the effect of CHO mouth rinse on performance after results demonstrated CHO ingestion improved performance during high-intensity exercise and was not accompanied by an increased CHO oxidation [4]. Furthermore, they showed the positive mouthwash effect was eliminated when glucose was infused instead ingested [3]. Together these results indicated that an oral CHO rinse may exert its effects during high-intensity exercise through a central action mediated by receptors in the mouth or GI tract, improving motor drive or motivation. Furthermore, CHO ingestion during high intensity exercise increases the potential incidence of gastrointestinal problems [5,6] and the CHO mouth rinse might be an alternative strategy to reduce any potentially debilitating incidence of gastrointestinal problems in endurance events lasting approximately one hour.

Several studies have reported that CHO mouth rinse improves both cycling [1,2] and running performance [7], and included different protocols to assess performance. For example, Carter *et al.* [2] reported a faster cycling time trial performance covering a set amount of work (914 ± 40 kJ, ~1 h TT), while Rollo *et al.* [7] found that CHO mouth rinse increased the distance covered during a time-based, running time trial, compared to placebo (PLA). Other studies that adopted similar exercise protocols also showed improvements in performance with CHO mouth rinse [1,8]. The mechanism by which CHO mouth rinse increases performance is not fully understood, but it may involve a group of receptors in the oral cavity with connections to the reward areas in the brain [9].

The activation of reward areas in the brain, such as the insula/frontal operculum, orbitofrontal cortex and striatum was suggested to lower perception of exertion during the exercise [1,10], and possibly reduce the feeling of displeasure [11]. However, some evidence suggests that the magnitude of performance improvements with CHO mouth rinse may be dependent on several factors, including duration of fasting [12] and time of mouth rinse [13]. While there is a growing number of publications about the effect of CHO mouth rinse on performance [1,2,8,13,14,15], no systematic review and quantitative measurement of the magnitude of CHO mouth rinse effect on performance has been performed. In order to investigate whether CHO mouth rinse significantly improves performance during high-intensity exercises lasting ~1 h, we conducted a systematic review of the literature coupled with a quantification of the overall mean difference across the studies. The main mechanisms involving the CHO mouth rinse were explored and main bias among the studies was also identified.

## 2. Methods

A search of all articles up to May 2013, which have investigated the effect of CHO mouth rinse on performance, were examined with no publication date or language limits. The search encompassed the following electronic databases: Pubmed (National Library of Medicine U.S.), SciELO (Scientific Electronic Library Online), Science Direct, LILACS, MEDLINE (International Literature on Health Sciences), and the Cochrane Library (Cochrane Central Register of Controlled Trials). The following search terms were used: carbohydrate combined with mouth and rinse. We used the logical operator “and” to combine the descriptors. Original articles conducted in humans were considered, and any articles that reported CHO intake with no specific mouthwash protocol were excluded.

The systematic review procedures consisted of four steps: (1) to read the titles of the studies; (2) to verify duplication; (3) to read the papers fully; and (4) to check for exclusion criteria carried out by three independent researchers and complete a double check on reference lists. Each study was further categorized referring to authors, year of publication (reference), type of activity/exercise protocol, sample size (*n*), level of performance, duration of fasting, experimental design, mouth rinse protocol, solutions offered, results, and main conclusions.

In addition, the individual and overall mean differences between PLA and CHO mouth rinse were calculated in seven of the eleven eligible studies that reported power output (W) as the main performance variable. As power output was the most reported outcome, an overall mean difference analysis using mean power output was chosen instead of variables such as time to completion for a given distance or work, time to exhaustion, or distance covered in a given time.

## 3. Results

Following an initial search of the database, one hundred and thirty-two publications were identified as potentially eligible for inclusion. Twenty-eight articles remained following a titles analysis (step 1). Eliminating duplicity (step 2), and subsequent application of the exclusion criteria (step 4), eight studies were deemed appropriate. However, three more articles were identified from the reference lists of these studies and considered eligible for inclusion. In the total, eleven articles were eligible for this review. The characteristics and main results from reviewed studies are displayed in Table 1.

**Table 1:** Summary of the studies investigating the effect of carbohydrate mouth rinse on performance during exercise.

Reference	Type of activity/exercise protocol	Sample (*n*)	Fast (h)	Design	Duration of mouth rinse/beverage concentration (%)	Number of mouth rinses	Distinguish between the solutions ***	Main results (mean ± SD)	(% Enhanced Performance)
Chambers *et al.* [1]	Cycling Time-trial ~1 h (914 ± 29 kJ) ~75% W_max_	8 M (ET)	6	Double-Blinded	10 s/Glucose (6.4%) *vs.* PLA (saccharin + aspartame in water: 150 mL/1000 mL)	8	0	Time (min) 60*.*4 *±* 3*.*7* vs.* 61*.*6 *±* 3*.*8	Yes, 1.99%
Chambers *et al.* [1]	Cycling Time-trial ~1 h (914 ± 29 kJ) ~75% W_max_	6 M and 2 W (ET)	6	Double-Blinded	10 s/MALT (6.4%) + saccharin and aspartame *vs.* PLA (saccharin and aspartame in water: 150 mL/1000 mL)	8	0	Time (min) 62.6 ± 4.7 *vs.* 64.6 ± 4.9	Yes, 3.19%
Carter *et al.* [2]	Cycling Time-trial ~1 h (~914 ± 40 kJ) ~75% Wmax	7 M and 2 W (ET)	4	Blinded	5 s/MALT (6.4%) *vs.* Water	8	4 (4)	Time (min) 59.6 ± 0.5 *vs.* 61.4 ± 0.5	Yes, 3.02%
Rollo *et al.* [7]	Running Time-trial 30 min ~60% VO_2_max	10 * (ET)	Overnight fast	Double-Blinded	5 s/CHO (6%) *vs.* PLA	9	2 (**)	Distance (m) 6584 *±* 520* vs.* 6469 *±* 515	Yes, 1.78%
Pottier *et al.* [8]	Cycling Time-trial ~1 h (975 ± 85 kJ) ~75% W_max_	12 * (ET)	3	Double-Blinded	5 s/CHO-E (6%) *vs.* PLA	8	**	Time (min) 61*.*7 *±* 5*.*1* vs.* 64*.*1 *±* 6*.*5	Yes, 3.89%
Pottier *et al.* [8]	Cycling Time-trial ~1 h (975 ± 85 kJ) ~75% W_max_	12 * (ET)	3	Double-Blinded	Ingestion CHO-E (6%) *vs.* PLA	8	**	Time (min) 63.2 *±* 6.9 *vs.* 62.5 *±* 6.9	No, −1.11%
Beelen *et al.* [12]	Cycling Time-trial ~1 h (1.053 ± 48 kJ) ~75% W_max_	14 M (ET)	2	Double-Blinded	5 s/MALT (6.4%) *vs.* Water	8	5 (4)	Time (min) 68*.*1 *±* 0.3* vs.* 67*.*5 *±* 0.3	No, −0.91%
Sinclair *et al.* [13]	Cycling time trial 30-min	11 M	4	Blinded	5 s/MALT (6.4%) *vs.* Water	5	11 (5)	Power Output (W) 153 ± 17 *vs.* 146 ± 13	Yes, 4.34%
Sinclair *et al.* [13]	Cycling time trial 30-min	11 M	4	Blinded	10 s/MALT (6.4%) *vs.* Water	5	11 (6)	Power Output (W) 156 ± 17 *vs.* 146 ± 13	Yes, 6.36%
Fares and Kayser [14]	Cycling ~60% W_max_ until exhaustion	13 M (NA)	3	Blinded	5–10 s/CHOFS (6.4%) *vs.* PLAFS (water)	12	8 (4)	Time (min) 56.6 ± 12.2 *vs.* 54.7 ± 11.3	Yes, 3.47%
Fares and Kayser [14]	Cycling ~60% W_max_ until exhaustion	13 M (NA)	Overnight fast	Blinded	5-10 s/FCHO (6.4%) * vs.* FPLA (water)	12	7 (4)	Time (min) 53.9 ± 12.8 *vs.* 48.3 ± 15.3	Yes, 11.59%
Rollo *et al.* [15]	Running Time-trial ~1 h ~60% VO_2max_	10 M (ET)	~14	Double-Blinded	5 s/CHO-E (6.4%, mouth rinse without intake) *vs.* PLA (mouth rinse + intake)	4	**	Distance (m) 14283 *±* 758 *vs.* 14190 *±* 800	No, 0.65%
Rollo *et al.* [15]	Running Time-trial ~1 h ~60% VO_2max_	10 M (ET)	~14	Double-Blinded	5 s/CHO-E (6.4%, mouth rinse + intake) *vs.* PLA (mouth rinse + intake)	4	**	Distance (m) 14515 ± 756 *vs.* 14190 ± 800	Yes, 2.29%
Whitham and Mckinney [16]	Running Time-trial 45 min (1.053 ± 48 kJ) ~65% VO_2max_	7 M (RA)	4	Double-Blinded	5 s/ MALT (6% maltodextrin-97% polysaccharide, 2% disaccharide, 1% glucose + 3% lemon juice) *vs.* PLA (3% lemon juice)	10	1 (1)	Distance (m) 9333 *±* 988 *vs.* 9309 *±* 993	No, 0.26%
Rollo *et al.* [17]	Running Time-trial ~1 h ~60% VO_2max_	20 M (ET)	~14	Double-Blinded	5 s/CHO-E (6.4%) *vs.* PLA	4	0	Distance (m) 14298 ± 685 *vs.* 14086 ± 732	Yes, 1.50%
Lane *et al.* [18]	Cycling Time-trial ~1 h	12 M	Overnight fast	Double-Blinded	10 s/MALTFS (10%) *vs.* PLAFS (water)	9	** (3)	Power output (W) 286 ± 6 *vs.* 285 ± 1	Yes, 1.8%
Lane *et al.* [18]	Cycling Time-trial ~1 h	12 M	Overnight fast	Double-Blinded	10 s/FMALT (10%) * vs.* FPLA (water)	9	** (3)	Power output (W) 282 ± 6 *vs.* 273 ± 6	Yes, 3.4%

* No gender specification; ** Not reported; *** Number of distinguishing (number of correct distinguishing is given in parentheses). M—men; W—women; ET—endurance trained; RA—recreationally active; NA—nonathletic; CHO-E—electrolyte solution at carbohydrate; GLU—glucose; MALT—maltodextrin; PLA—placebo; FCHO—carbohydrate rinse in fasted state; FPLA—placebo in fasted state; CHOFS—carbohydrate rinse in fed state; PLAFS—placebo in fed state; MALTFS—maltodextrine rinse in fed state; FMALT—maltodextrine rinse in fast state.

### 3.1. Type of Activity/Exercise Protocol

The most common exercise protocols used in the studies were either cycle time trial with fixed total work (~1 h duration, intensity ~75% VO_2_max) or running time trial (~1 h duration, intensity between 60% and 65% VO_2_max). One study performed time to exhaustion test to measure performance. Eight articles were double-blinded, while three studies were only single blinded.

### 3.2. Sample

The sample size (*n*) in any one study ranged from seven to sixteen individuals, totaling one hundred and thirty-four volunteers. Seven studies involved only males, two both genders, and two did not specify participant gender. A majority of the studies involved endurance trained volunteers, except in two studies in which participants were either moderately trained or untrained. The duration of fasting prior to the testing ranged between two and 15 h.

### 3.3. Mouth Rinse Protocols

There was a large variation in mouth rinse protocols between the studies, including: (1) duration of mouth rinse (5 or 10 s); (2) mouth rinse repetitions during the performance trial (4 to 12 times); and (3) solution (maltodextrin, lemon juice, glucose, artificial sweeteners, and saccharin). In addition, CHO solution was either mouth rinsed and expectorated (*n* = 10) or subsequently ingested (*n* = 1). In two studies, the volunteers were not able to distinguish the CHO mouth rinse solution. In addition, the volunteers noticed differences between CHO and PLA solutions in seven studies but only in two cases were able to distinguish correctly. Two studies did not report solution differentiation assessment.

### 3.4. Performance

**Figure 1:**
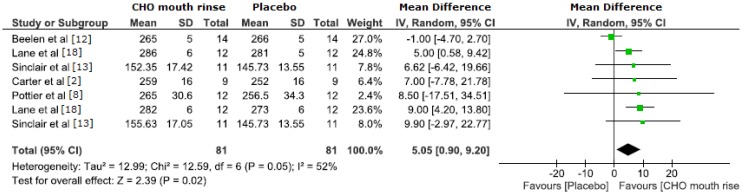
The overall effect of CHO mouth rinse on power output (W) as the main performance outcome.

Eight of the eleven eligible studies found an improvement in exercise performance (decreased time to complete the time-trial, increased running distance, or increased time to exhaustion) with CHO mouth rinse (glucose or maltodextrin) (Table 1). However, one study reported that CHO mouth rinse influenced performance only when followed by ingestion [15], while another one found that mouth rinse, but not ingestion of CHO solution, had an effect on performance [8]. Three studies found no effect of CHO mouth rinse on performance [12,15,16]. Two of these studies used running to access performance [15,16]. Power output was the most reported outcome (seven studies), so an overall mean difference analysis using mean power output was performed. The overall effect of CHO mouth rinse on performance was significant with a mean difference of 5.05 W (95% CI 0.90 to 9.2 W, *z* = 2.39, *p* = 0.02). However, there was a large intra and inter study variability observed (*I*^2^ = 52%), as displayed in Figure 1.

### 3.5. Rating of Perceived Effort

The rating of perceived exertion (RPE) did not differ between CHO and PLA mouth rinse in eight studies, while two studies did not report the RPE, and one study reported reduction in RPE with CHO mouth rinse. The brain areas activated by the CHO mouth rinse (glucose and maltodextrin) were investigated in only one study, and it was found that brain regions associated with reward, including insula/frontal operculum, orbitofrontal cortex, and striatum were significantly activated [1].

## 4. Discussion

The present review identified eleven studies investigating the influence of CHO mouth rinse on endurance exercise performance. A majority of these studies reported improved performance with either glucose or maltodextrin mouth rinses [1,2,7,8,13,14,17,18]. The pioneering study investigating CHO mouth rinse was conducted by Carter *et al.* [2], who found an improvement in the exercise performance when CHO was subsequently expelled and not ingested. The ergogenic effect derived from CHO mouth rinse does not seem to be a result of its absorption, as it has been reported that CHO mouth rinse is not associated with changes in blood glucose concentration [7,15,17].

Two studies did not find a beneficial effect of CHO mouth rinse on performance [12,16], while in one study the effect of mouth rinse was apparent when followed by ingestion [15]. It is noteworthy that participants in the first two studies [12,16] performed the exercise in a postprandial state. It has been suggested that the prior fasting period may be required for a positive effect of mouth rinse, suggesting that potential benefit of CHO mouth rinse is, at least partially, dependent on endogenous CHO (liver and muscle glycogen) stores [12]. Lane and colleagues [18] concluded that a CHO mouth rinse improves performance to a greater extent in a fasted compared with a fed state. Another study [14] found that CHO mouth rinse improved time to exhaustion in both pre (overnight fast) and postprandial (3 h after meal) states. However, individuals performed the trial listening to music in that study which may have masked the influence of fasting, as it has been shown that listening to music, *per se*, can improve performance [19]. Otherwise, other studies having participants fasting 4 h or less [2,8,13,14,18] found a beneficial effect of CHO mouth rinse on performance, suggesting that other factors than duration of fasting may have influenced the absence of CHO mouth rinse effect in some studies [12,16].

It has also been demonstrated that the sweetness of CHO does not influence the level of activation of the oral receptors. Glucose is a simple CHO with a sweet taste while maltodextrin is a complex CHO and tasteless. However, Chambers [1] revealed that both glucose and maltodextrin increased similarly the performance and insula/frontal operculum, orbitofrontal cortex, and striatum activation [1]. This indicates there may be a class of unidentified oral receptors that responds to CHO content of the solution independently of sweetness. Interestingly, these brain areas are associated with reward, which probably leads to an increase in the exercise intensity mediated by a reduction in the perceived exertion and an increased pleasure.

The RPE was not different between PLA and CHO mouth rinses in eight studies and indicates that participants were able to produce more power for a given RPE in the CHO mouth rinse condition. Similarly, in the only study investigating the CHO mouth rinse on a constant-load exercise [14], RPE was reduced in CHO mouth rinse compared to PLA condition. The pathway by which reward areas in the brain are activated remains to be clarified further, but it seems to be plausible that rinsing the mouth with a CHO solution activates the chemoreceptors on the tongue and oral cavity, exciting first-order neurons that carry information to the Rostral Nucleus of the Solitary Tract (rNTS) [9]. The rNTS probably acts on the ventral posterior medial nucleus of the thalamus (VPMpc) neurons projecting to the insular cortex. The insular cortex could stimulate the motor cortex excitability, reducing RPE and influence any motor neural feedback to increase the power output during the exercise [9]. Additionally, the CHO mouth rinse may induce an increased pleasure via the lamina I spinothalamocortical system, which seems to influence interception and modification of neural feedback involved with emotion and motivation [20].

We also found that six of the seven articles reporting power output as the main outcome had a positive main effect favoring CHO mouth rinse (Figure 1). However, the calculated 95% CI was large and overlapped zero in four of these six studies. Similarly, even with an overall mean difference significantly favoring CHO mouth rinse, there was a large intra and inter study variability of CHO mouth rinse effect on power output. The large variability in these studies suggests that methodological factors should be considered and better controlled/reported, including: (1) duration of fasting; (2) duration and number of mouth rinse; (3) solution concentration; (4) type of activity and exercise protocol; (5) sample size; (6) intervenient factors (e.g., listening to music and muscle and liver glycogen levels before the trial). In particular, there is evidence pointing that a 10-s mouth rinse may be better than a 5 s mouth rinse on performance, suggesting a dose response to the duration of mouth rinse [13]. In addition, we observed a large range in the number of mouth rinses during the performance trial between the studies (from 4 to 12 times), but no study investigated if a higher number and/or a shorter interval between CHO mouth rinses would result in an improved performance. Therefore more studies using standardized protocols and larger sample sizes are necessary to ascertain both the effect of CHO mouth rinse on performance and its mechanism of action. Further studies should also investigate different forms of “placebo”, e.g., water *versus* no water *versus* artificially flavored fluids. Recent evidence indicates that repeated mouth rinsing with water results in decreased performance relative to not rinsing at all, suppressing partially the CHO mouth rinse effect [21]. 

## 5. Conclusions

CHO mouth rinse seems to improve performance during moderate- to high-intensity exercise (~60% to 75% VO_2_max), of at least 1 h duration. It is probable that the mechanism involved in this improvement may not be metabolic but neural, via oral CHO receptors (glucose and maltodextrin) that activate brain regions related to the sensation of reward and pleasure. These receptors appear to be especially responsive in metabolic conditions of reduced endogenous CHO stores (muscle and liver glycogen), but further investigation is required. The CHO mouth rinse might be an alternative to the intake, avoiding any potentially performance debilitating incidence of gastrointestinal problems when CHO is ingested during high-intensity exercise or during competitions lasting ~1 h. Thus, athletes with historically problematic CHO induced GI issues may be benefited for CHO mouth rinse. However, the precise identification of the oropharyngeal receptors, the mechanism of activation of the brain regions, as well as more standardized and controlled protocols are necessary to clarify the mechanism and magnitude in which the CHO mouth rinse promotes improvement in the performance.

It is prudent to also point out that spitting out CHO/fluid replacement drink may compromise energy substrate supply, hydration and blood glucose maintenance and jeopardize performance during events lasting longer than 1 h. Therefore, further investigation for performance over longer duration is required.
